# A novel mitochondrial micropeptide MPM enhances mitochondrial respiratory activity and promotes myogenic differentiation

**DOI:** 10.1038/s41419-019-1767-y

**Published:** 2019-07-11

**Authors:** Yi-Fang Lin, Man-Huan Xiao, Hua-Xing Chen, Yu Meng, Na Zhao, Liang Yang, Haite Tang, Jia-Lei Wang, Xingguo Liu, Ying Zhu, Shi-Mei Zhuang

**Affiliations:** 1 MOE Key Laboratory of Gene Function and Regulation, School of Life Sciences, Collaborative Innovation Center for Cancer Medicine, Sun Yat-sen University, Xin Gang Xi Road 135, 510275 Guangzhou, China; 2 CAS Key Laboratory of Regenerative Biology, Hefei Institute of Stem Cell and Regenerative Medicine, Guangzhou Regenerative Medicine and Health Guangdong Laboratory, Guangdong Provincial Key Laboratory of Stem Cell and Regenerative Medicine, Institute for Stem Cell and Regeneration, Guangzhou Institutes of Biomedicine and Health, University of Chinese Academy of Sciences, Chinese Academy of Sciences, 510530 Guangzhou, China; 3 Key Laboratory of Liver Disease of Guangdong Province, The Third Affiliated Hospital, Sun Yat-sen University, 510630 Guangzhou, China

**Keywords:** Peptides, Cell signalling

## Abstract

Micropeptides belong to a class of newly identified small molecules with <100 amino acids in length, and their functions remain largely unknown. Here, we identified a novel muscle-enriched micropeptide that was localized to mitochondria (named MPM, micropeptide in mitochondria) and upregulated during in vitro differentiation of C2C12 myoblasts and in vivo early postnatal skeletal muscle development, and muscle regeneration after cardiotoxin (CTX) damage. Downregulation of MPM was observed in the muscular tissues of tibial muscular dystrophy and Duchenne muscular dystrophy patients. Furthermore, MPM silencing inhibited the differentiation of C2C12 myoblasts into myotubes, whereas MPM overexpression stimulated it. MPM^−/−^ mice exhibited smaller skeletal muscle fibers and worse muscle performance, such as decrease in the maximum grip force of limbs, the latency to fall off rotarod, and the exhausting swimming time. Muscle regeneration was also impaired in MPM^−/−^ mice, as evidenced by lower expression of Pax7, MyoD, and MyoG after CTX injection and smaller regenerated myofibers, compared with wild-type mice. Mechanistical investigations based on both gain- and loss-of function studies revealed that MPM increased oxygen consumption and ATP production of mitochondria. Moreover, ectopic expression of PGC-1α, which can enhance mitochondrial respiration, attenuated the inhibitory effect of siMPM on myogenic differentiation. These results imply that MPM may promote myogenic differentiation and muscle fiber growth by enhancing mitochondrial respiratory activity, which highlights the importance of micropeptides in the elaborate regulatory network of both myogenesis and mitochondrial activity and implicates MPM as a potential target for muscular dystrophy therapy.

## Introduction

Recent studies have revealed that many small open reading frames (sORFs) may encode micropeptides with <100 amino acids in length^1^. Emerging evidences suggest that micropeptides may act as important regulators in fundamental biological processes, such as metabolism^2^, cell death^3,4^, and development^5,6^. However, their functions remain largely unexplored.

Myogenesis is an important event required for muscle development and regeneration, and its dysfunction may cause severe muscle diseases, such as Duchenne muscular dystrophy (DMD) and tibial muscular dystrophy (TMD)^7–10^. Myogenesis is a highly coordinated process, including activation, proliferation, and differentiation of muscle progenitor cells into fused, multinucleated myotubes. Myogenic differentiation is the fundamental step during myogenesis and is regulated by numerous proteins and noncoding RNAs. After activation of Pax7, the expression of the master transcription regulatory factors of myogenic differentiation, including MyoD and Myf5, is upregulated, followed by the activation of other myogenic regulatory factors (MRFs), such as MyoG^11,12^. A few noncoding RNAs, like miR-31 and linc-RAM^13,14^, are also shown to regulate MRFs. To date, only two micropeptides have been reported to regulate myogenic differentiation^15–19^. Obviously, more extensive investigations are required to identify more myogenesis-related micropeptides.

In this study, we found that micropeptide in mitochondria (MPM), a muscle-enriched and mitochondria-localized micropeptide, was upregulated during myogenic differentiation in vitro and in vivo, and promoted differentiation of C2C12 myoblasts into myotubes. MPM^−/−^ mice exhibited smaller skeletal muscle fibers, worse muscle performance, and compromised muscle regeneration. Mechanistically, MPM may promote myogenic differentiation by enhancing mitochondrial respiratory activity. These findings extend our understanding of the biological function of micropeptides and the regulation of myogenesis.

## Results

### A novel muscle-enriched micropeptide is localized to mitochondria and promotes myogenic differentiation

**Fig. 1:**
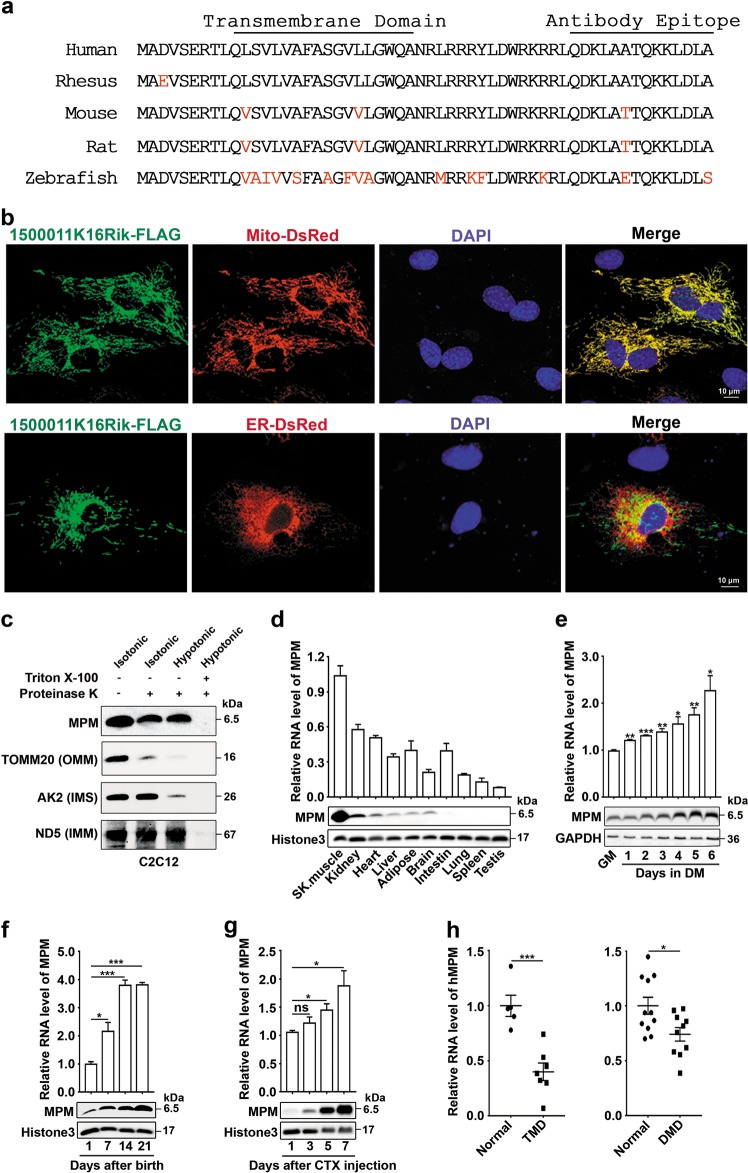
MPM is located in mitochondria and upregulated during myogenic differentiation. **a** Amino acid sequence alignment of vertebrate MPM proteins. The amino acids in red letters indicate the non-perfectly conserved amino acids. **b**, **c** MPM was localized to the inner mitochondrial membrane. For **b**, immunofluorescent staining for FLAG (green) and the marker of mitochondria (Mito-DsRed, red) (*upper*) or endoplasmic reticulum (ER-DsRed, red) (*lower*) in 1500011K16Rik-FLAG-transfected C2C12 myoblasts. Scale bar, 10 μm. For **c**, mitochondria were isolated from C2C12 myoblasts, subjected to proteinase K digestion in isotonic or hypotonic buffer and then western blotting. TOMM20, AK2, and ND5 were used as the controls for the outer mitochondrial membrane (OMM) protein, the intermembrane space (IMS) protein, and the inner mitochondrial membrane (IMM) protein, respectively. **d** MPM was highly abundant in mouse skeletal muscle. MPM expression was detected in the indicated tissues from 6-week-old male mice (*n* = 6). **e** MPM expression was significantly increased during differentiation of C2C12 myoblasts. C2C12 cells were cultured in 10% FBS-containing DMEM (GM) to reach 90% confluence and then incubated in differentiation medium (DM) for 1–6 days. **f** MPM levels were increased in mouse muscle during postnatal development. The gastrocnemius muscles were collected from mice (*n* = 3) at the indicated time. **g** MPM expression was increased during skeletal muscle regeneration. CTX was injected into the left gastrocnemius muscle of 5-week-old C57BL/6 male mice (*n* = 3). The right gastrocnemius muscles injected with NaCl were served as control. Then the damaged muscles were harvested at the indicated time. **h** hMPM RNA level was downregulated in the muscular tissues of patients with tibial muscular dystrophy (TMD) or Duchenne muscular dystrophy (DMD). The expression data were derived from GEO datasets (GSE42806 and GSE1007). The mean value of hMPM RNA level in normal control was set as 1. For **d**–**g**, the RNA and protein levels of MPM were detected by qPCR (*upper*) and western blotting (*lower*). GAPDH and histone 3 were used as the internal control for cell line and tissue, respectively. For **d–h**, the data are expressed as the mean ± SEM. *P < 0.05; **P < 0.01; ***P < 0.001; ns, not significant

To identify novel micropeptides associated with myogenic differentiation, a bioinformatics analysis was conducted based on mouse muscle transcriptome profile and SWISS PROT data. Among 3853 transcripts detected in mouse skeletal muscle, 162 transcripts were predicted to have micropeptide-coding potential, and 25 of them had not been reported yet. Five out of these twenty-five transcripts were upregulated during differentiation of C2C12 myoblasts into myotubes, and mouse 1500011K16Rik (LINC00116 in human), the one with the most abundant expression in the muscle (Supplementary Fig. 1a, b and Supplementary Table 1), was selected for further exploration. The 5′- and 3′-RACE assays revealed that mouse 1500011K16Rik had two RNA transcript isoforms, and the 447-bp transcript was more abundant than the 811-bp transcript. Human LINC00116 had only one 442-bp transcript (Supplementary Fig. 2a, b). These transcripts were predicted to encode a single-pass transmembrane micropeptide that contained 56 amino acids and was highly conserved across various vertebrate species (Fig. 1a and Supplementary Fig. 2a, b).

To validate the coding capacity of 1500011K16Rik/LINC00116, an expression construct, comprising 447-bp 1500011K16Rik or 442-bp LINC00116 transcript (Supplementary Fig. 2a, b) with a FLAG epitope tag in-frame appended to the C-terminus of the coding sequence, was then transfected into cells. A ~7.5 kDa peptide, corresponding to the predicted molecular weight, was detected in the transfectants by anti-FLAG antibody (Supplementary Fig. 3a), demonstrating existence of 1500011K16Rik/ LINC00116*-*encoded micropeptide. Furthermore, this micropeptide was colocalized with mitochondrial targeted-DsRed (Mito-DsRed) but not endoplasmic reticulum targeted-DsRed (ER-DsRed) (Fig. 1b), which was therefore named MPM. Consistently, endogenous MPM was detected using anti-MPM, a customized polyclonal antibody raised against the C-terminal region of MPM (Fig. 1a and Supplementary Fig. 3b). Anti-MPM detected a strong band with expected size in the cells transfected with vector containing sORF of mouse MPM (171 bp, Supplementary Fig. 2a), but no expected band was detected when 1-base pair deletion was generated in the second codon of sORF (MPM-FS, Supplementary Fig. 3c). Consistently, endogenous MPM was cofractionated with voltage dependent anion channel 1 (VDAC1), a well-known mitochondrial protein (Supplementary Fig. 4). Further investigations revealed that MPM exhibited a proteolysis pattern similar to the inner mitochondrial membrane protein ND5, suggesting the localization of MPM in the inner mitochondrial membrane (Fig. 1c).

Next, analyses on a panel of adult mouse tissues confirmed the existence of cellular MPM and revealed that MPM was highly abundant and enriched in skeletal muscle (Fig. 1d). Moreover, both RNA and protein levels of MPM significantly increased during the processes of C2C12 differentiation (Supplementary Fig. 5 and Fig. 1e) and mouse postnatal skeletal muscle development (Fig. 1f), and along with the progress of mouse muscle regeneration after cardiotoxin (CTX) damage (Fig. 1g). Interestingly, the RNA level of human MPM was downregulated in the muscular tissues of patients with TMD or DMD (Fig. 1h), which were partially due to deficiency in the proliferation and differentiation capacity of satellite cells and myoblasts^8,9^. These data suggest that MPM is important for myogenic differentiation.

**Fig. 2:**
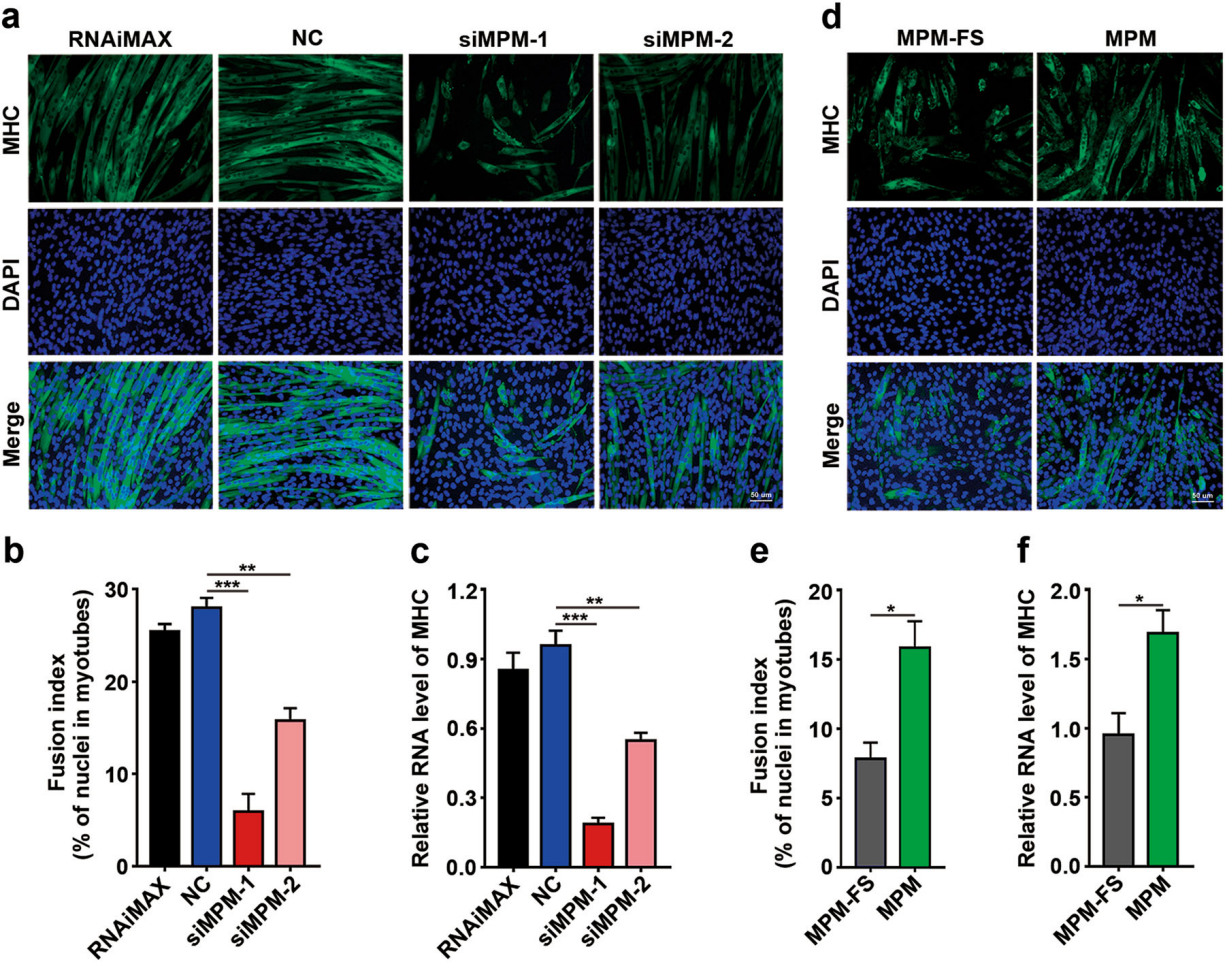
MPM promotes myogenic differentiation of C2C12 myoblasts. **a**–**c** Silencing MPM decreased the fusion index of myotubes and the RNA level of MHC. C2C12 myoblasts were transfected twice at 24 h intervals with the indicated RNA duplexes. Twenty-four hours after the last transfection, the cells were refreshed with differentiation medium and incubated for 72 h, then subjected to immunofluorescence staining (**a**, **b**) or qPCR (**c**) assays. RNAiMAX, cells exposed to Lipofectamine RNAiMAX without RNA duplexes. **d**–**f** Ectopic MPM expression increased the fusion index of myotubes and the RNA level of MHC. Twenty-four hours after transfection with the indicated plasmids, C2C12 cells were reseeded in 10% FBS-containing DMEM for 24 h, followed by culture in differentiation medium for 48 h before immunofluorescence staining (**d**, **e**) or qPCR assays (**f**). MPM-FS, MPM with 1-base pair deletion in the second codon, were used as negative control. For **a**, **d**, MHC was stained green, and cell nuclei were stained blue with DAPI. Scale bar, 50 μm. For **b**, **e**, the percentage of nuclei in myotubes is presented as fusion index. For each sample, 1000 nuclei were counted. For **b**, **c**, **e**, **f**, error bars represent mean ± SEM from three independent experiments. *P < 0.05; **P < 0.01; ***P *<* 0.001

The regulatory role of MPM was further verified based on loss- and gain-of-function studies. MPM silencing by two siRNAs, siMPM-1 and siMPM-2 (Supplementary Fig. 3b), significantly attenuated the differentiation of C2C12 myoblasts into myotubes, as evidenced by a reduced fusion index of myotubes and decreased level of myosin heavy chain (MHC) (Fig. 2a–c). Contrarily, overexpression of MPM (Supplementary Fig. 3c) significantly enhanced C2C12 differentiation (Fig. 2d–f), suggesting the promoting role of MPM in myogenic differentiation.

### MPM^−/−^ mice exhibit smaller skeletal muscle fibers, worse muscle performance, and compromised skeletal muscle regeneration

**Fig. 3:**
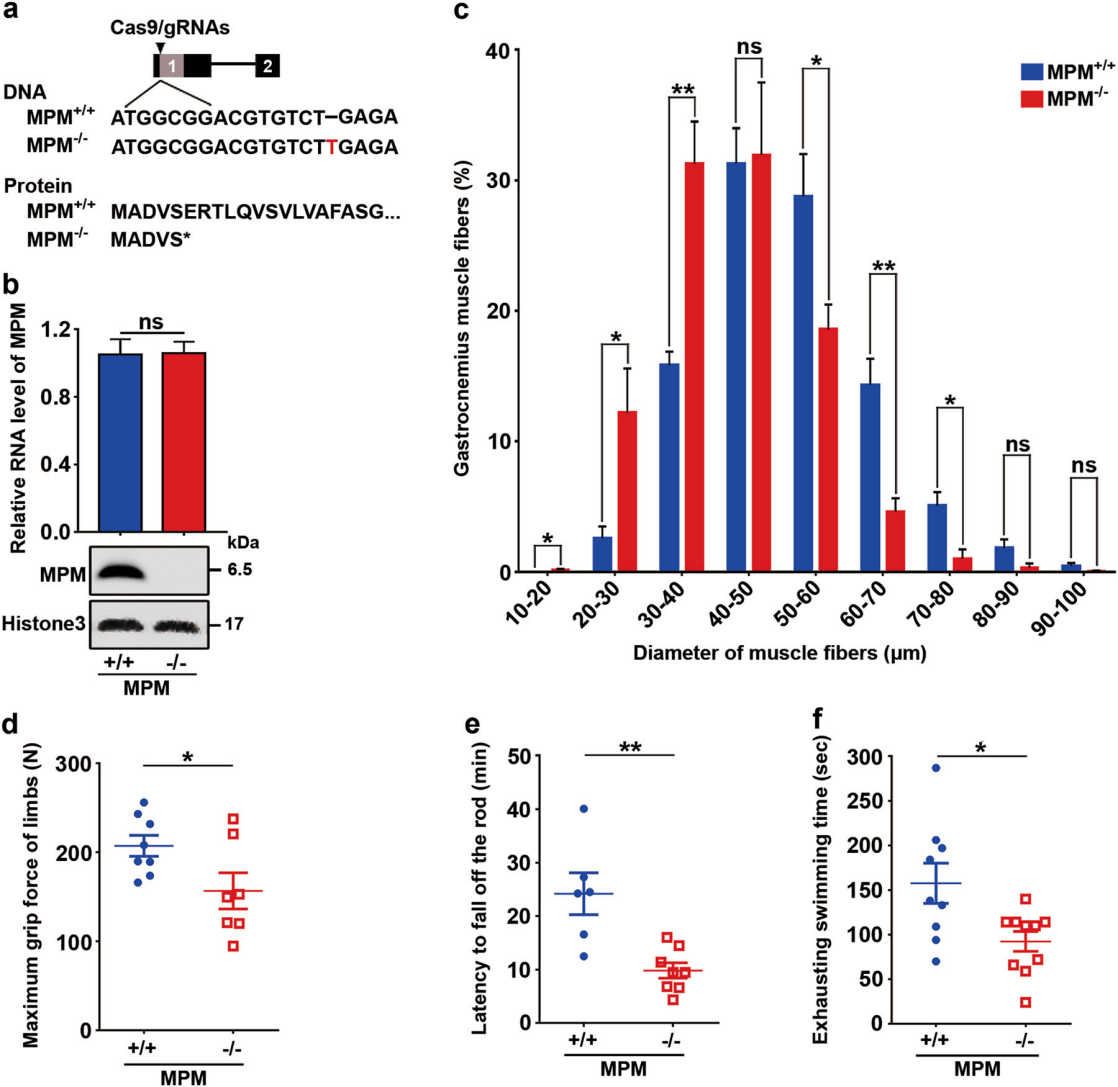
MPM^−/−^ mice exhibit smaller skeletal muscle fibers and worse muscle performance. **a** MPM knockout mouse was generated by 1-base pair insertion (red letter) which created a premature stop codon after codon 5. **b** MPM protein but not mRNA was eliminated in the muscle of MPM^−/−^ mice. The RNA and protein levels of MPM in the skeletal muscle of 6-week-old MPM^+/+^ (*n* = 5) and MPM^−/−^ (*n* = 5) male mice were detected by qPCR (*upper*) and western blotting (*lower*). **c** MPM^−/−^ mice had smaller gastrocnemius myofibers than MPM^+/+^ mice. The diameters of myofibers from MPM^−/−^ (*n* = 4) and MPM^+/+^ (*n* = 4) mice are shown. **d** MPM^−/−^ mice showed decrease in the maximum grip force of limbs. Seven MPM^−/−^ mice and eight MPM^+/+^ mice were examined. **e** MPM^−/−^ mice showed worse rotarod performance. The latency of mice to fall off the rotarod was recorded for MPM^+/+^ (*n* = 6) and MPM^−/−^ mice (*n* = 8). **f** MPM^−/−^ mice (*n* = 10) had shorter exhausting swimming time than MPM^+/+^ mice (*n* = 9). Six- to eight-week-old male mice were used in **c**–**f** and data are presented as mean ± SEM in **b**–**f**. *P < 0.05; **P < 0.01; ns, not significant

To further assess the causative function of MPM in myogenic differentiation, the MPM knockout mouse was generated by using the clustered regularly interspaced short palindromic repeats (CRISPR)-associated protein 9 (cas9) system to disrupt the coding frame of MPM. A founder with 1-base pair insertion that created a premature stop codon after codon 5 (MPM^−/−^, Fig. 3a) was chosen for further analysis. As expected, MPM protein was eliminated in the muscle of MPM^−/−^ mice, while the mRNA level of MPM was similar between MPM^+/+^ and MPM^−/−^ mice (Fig. 3b). MPM^−/−^ mice were born at the expected Mendelian frequency. Compared with MPM^+/+^ mice, MPM^−/−^ mice showed smaller myofibers in gastrocnemius (Fig. 3c) and worse muscle performance, including decrease in the maximum grip force of limbs (Fig. 3d), the latency to fall off the rotarod (Fig. 3e), and the exhausting swimming time (Fig. 3f). However, there was no difference in fertility, body weight, oral temperature, gait, sensitivity of pain, and unconditioned reflexes between MPM^+/+^ and MPM^−/−^ mice (Supplementary Fig. 6a–h).

**Fig. 4:**
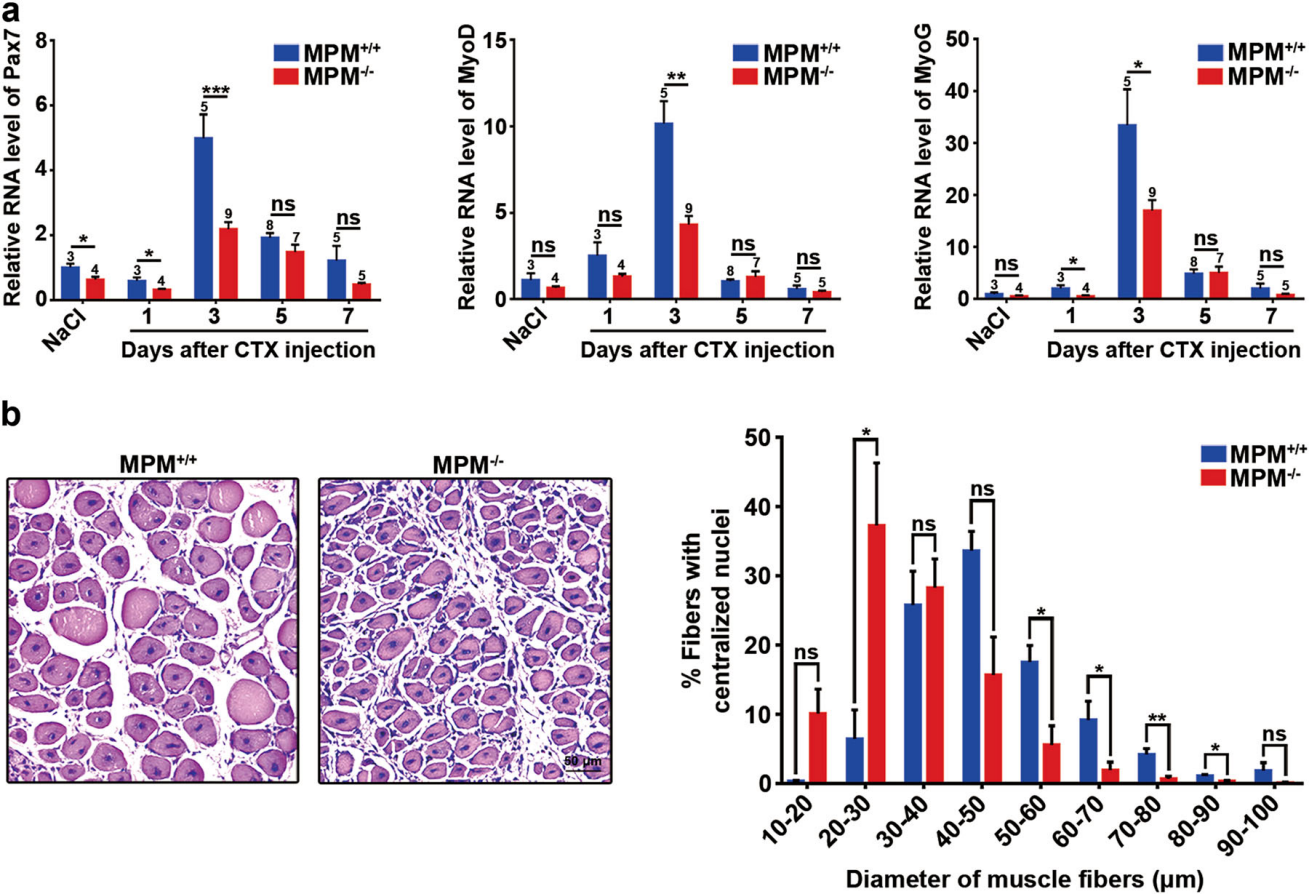
MPM knockout attenuates skeletal muscle regeneration after CTX treatment. **a** The RNA levels of Pax7, MyoD, and MyoG in the gastrocnemius muscles of MPM^+/+^ and MPM^−/−^ mice after injection with NaCl (control) or CTX. The number of mice used in each group is indicated over the bar. **b** MPM^−/−^ mice (*n* = 5) had smaller regenerated muscle fibers compared with MPM^+/+^ mice (*n* = 3). Representative images of H&E staining (*left* and *middle* panels, scale bar, 50 μm) and the diameters (*right* panel) of the regenerated myofibers with centralized nuclei at 7 days post CTX injection are shown. Five-week-old male mice were used and data are presented as mean ± SEM. *P *<* 0.05; **P *<* 0.01; ***P *<* 0.001; ns, not significant

The function of MPM in skeletal muscle regeneration was then investigated by treating MPM^+/+^ and MPM^−/−^ mice with CTX. As shown, the key myogenic transcription factors, including Pax7, MyoD, and MyoG, were significantly upregulated in both MPM^+/+^ and MPM^−/−^ mice at day 3 after CTX injection, with less increase in MPM^−/−^ mice (Fig. 4a). Moreover, the regenerated muscle fibers in MPM^−/−^ mice were significantly smaller than those in MPM^+/+^ mice (Fig. 4b), indicating that MPM knockout results in impaired satellite cell differentiation and muscle regeneration in vivo.

Taken together, these in vivo assays suggest that MPM may promote myogenic differentiation and thereby affect muscle growth and regeneration.

### MPM enhances mitochondrial respiratory activity to promote C2C12 myogenic differentiation

**Fig. 5:**
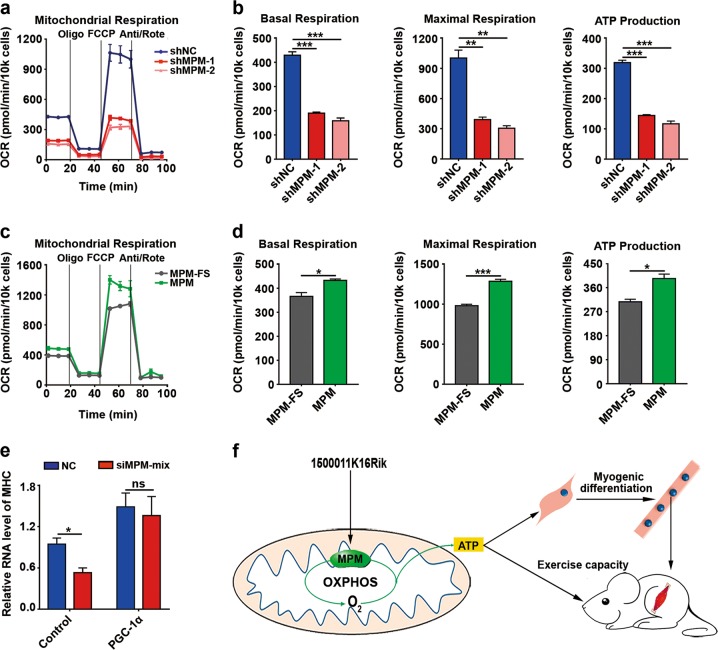
MPM enhances mitochondrial respiration to promote C2C12 myogenic differentiation. **a**, **b** MPM silencing inhibited mitochondrial respiratory activity. **c**, **d** MPM overexpression enhanced mitochondrial respiratory activity. For **a**–**d**, C2C12 myoblasts were transfected with the indicated plasmids for 48 h before detection for oxygen consumption rate (OCR). The basal and maximal oxygen consumption and ATP production of mitochondria (**b**, **d**) were calculated based on the OCR (**a**, **c**) detected by Seahorse XFe24 analyzer. **e** PGC-1α overexpression attenuated the inhibitory effect of siMPM on myogenic differentiation. C2C12 myoblasts were transfected with the indicated plasmids for 12 h, then transfected twice at 12-h intervals with NC or a mixture of siMPM-1 and -2 (siMPM-mix). Twelve hours after the last transfection, the cells were refreshed with differentiation medium and incubated for 48 h, then subjected to qPCR assay for MHC level. Data are presented as mean ± SEM in **a**–**e**. *P < 0.05; **P < 0.01; ***P < 0.001; ns, not significant. **f** Working model of MPM function in myogenic differentiation

Mitochondrion is the primary organelle to produce energy that is critical for myogenic differentiation and muscle contraction. Notably, MPM silencing decreased the basal and maximal oxygen consumption and the ATP production of mitochondria (Supplementary Fig. 7 and Fig. 5a, b), while MPM overexpression increased the basal and maximal oxygen consumption and ATP production (Fig. 5c, d). Furthermore, ectopic expression of PGC-1α, which can promote mitochondria biogenesis and enhance mitochondrial respiration^20^, attenuated the inhibitory effect of siMPM on myogenic differentiation (Fig. 5e and Supplementary Fig. 8). These results suggest that MPM may promote myogenic differentiation by enhancing mitochondrial respiration.

Taken together, our findings suggest that MPM, a muscle-enriched micropeptide, is localized to the inner mitochondrial membrane, enhances mitochondrial respiratory activity, and promotes myogenic differentiation, which facilitates muscle growth and regeneration (Fig. 5f).

## Discussion

Although a large number of micropeptides have been annotated, only a few of them have been characterized functionally. In this article, we identify a novel micropeptide MPM and its critical role in myogenic differentiation.

Myogenic differentiation is an important event during muscle development and regeneration. It is elaborately regulated by numerous proteins and noncoding RNAs, such as MyoD, MyoG, Mrf4, miR-1, miR-133, miR-17-92, lnc-MyoD, linc-MD1, and linc-RAM^14,21–27^. To date, two micropeptides have been reported to regulate myogenic differentiation. Myomixer that is localized to plasma membrane promotes myoblast fusion via interacting with myomaker, a membrane activator of myoblast fusion and muscle formation^16–19^. Another micropeptide SPAR is localized to lysosome and suppresses muscle regeneration by inhibiting amino acid-induced mTORC1 activation^15^. Here, we identified a MPM as a positive regulator of myogenic differentiation based on both in vitro and in vivo studies: (1) MPM was upregulated during C2C12 differentiation in vitro, and postnatal skeletal muscle development and muscle regeneration in vivo; (2) myogenic differentiation of C2C12 myoblasts into myotubes was blocked by MPM silencing and stimulated by MPM overexpression; (3) MPM-knockout mice exhibited smaller skeletal muscle fibers and impaired skeletal muscle regeneration. These findings integrate MPM into the elaborate regulatory network of myogenic differentiation. Deregulation of myogenic differentiation is a common feature of muscular disorders^7,9^. We found that MPM was downregulated in muscle tissues of TMD and DMD patients, whose satellite cells and myoblasts lost their proliferation and differentiation capability^7,9^. Furthermore, MPM-knockout mice exhibited smaller skeletal muscle fibers, worse muscle performance, and impaired muscle regeneration. Considering its important function in muscle cell differentiation, MPM may be used as a potential target for anti-muscular dystrophy therapy.

The decline in muscle mass and strength is a hallmark of the aging^28,29^. We found that the skeletal muscle of aging mouse had a decreased level of MPM (data not shown) and MPM^−/−^ mice exhibited significant decrease in the maximum grip force of limbs and rapid development of muscle fatigue. Bioinformatic analysis based on public data (GEO: GSE362 and GSE674) revealed that MPM was also downregulated in aging human skeletal muscles (data not shown). These data suggest that MPM downregulation may be associated with muscle aging. It has been reported that the resting and maximal rates of oxygen consumption decline with age when corrected for lean mass^30–32^, and exercise stimulates mitochondrial metabolism to stave off the effects of aging^33,34^. In this study, overexpression of MPM increased the resting and maximal rate of oxygen consumption and promoted myoblast differentiation, indicating the potential to explore MPM for anti-aging therapy.

It has been reported that mitochondria play a critical role in myogenic differentiation and muscle function^35–40^. Mitochondrial respiration-derived metabolites, such as ATP, ROS, and NAD^+^, which may activate mitochondria-to-nucleus retrograde signaling and influence the expression or activity of transcription factors that regulate various cell activities^41–45^. We found that silencing MPM inhibited mitochondrial respiration, decreased the levels of ATP, and myogenic transcription factors, such as Pax7, MyoD, and MyoG, subsequently hindered myogenic differentiation and muscle development. Moreover, overexpression of PGC-1α, which has been shown to augment mitochondrial biogenesis and mitochondrial respiration^20^, attenuated the inhibitory role of siMPM on myogenic differentiation. Considering that MPM was located in the inner mitochondrial membrane, it might interact with other inner mitochondrial membrane proteins, like respiratory complexes, and regulate mitochondrial respiration and subsequent myogenic differentiation. Muscle exercise capacity is determined by mitochondrial respiratory activity and the size of muscle fibers^39,40,46,47^. This study revealed that silencing MPM impeded mitochondrial respiration and ATP production, inhibited myogenic differentiation and muscle fiber growth, which may be responsible for the decreased muscle exercise capacity in MPM^−/−^ mice.

While this paper was in the process of submission, two studies reported that the 1500011K16Rik-encoded micropeptide was located in mitochondria, and regulated oxidative phosphorylation and fatty acid oxidation^48,49^. To our knowledge, we present the first report identifying MPM as a new promoter of myogenic differentiation, skeletal muscle development, and regeneration, which may provide potential target for anti-muscular dystrophy and anti-aging therapy.

## Materials and methods

More details are provided in Supplementary Materials and Methods.

### Cell lines and cell culture

Mouse myoblast cell line, C2C12 (ATCC, CRL-1772) and human cervix carcinoma line, Hela (ATCC, CCL­2) were cultured in Dulbecco’s modified Eagle’s medium (DMEM; Life Technologies, Gaithersburg, MD, USA) supplemented with 10% fetal bovine serum (FBS; Hyclone, Logan, UT, USA), 100 μg/mL penicillin, and 100 μg/mL streptomycin. To induce myoblast differentiation, 90% confluent C2C12 cells were incubated in differentiation medium (DM) consisting of DMEM supplemented with 2% horse serum (16050122, Gibico, Carlsbad, CA, USA) and refreshed with DM everyday for 6 days.

### RNA oligoribonucleotides and vectors

The siRNAs targeting different sites of the mouse or human MPM transcript (Supplementary Fig. 2a, b) were designated as siMPM-1 and siMPM-2 for mouse and sihMPM-1 and sihMPM-2 for human, and purchased from RiboBio Co. (Guangzhou, China). The negative control RNA duplex for siRNA is nonhomologous to any mouse or human genome sequence. Sequences of all RNA oligoes used in this study are listed in Supplementary Table 2.

The following plasmids were used: pCDH-1500011K16Rik-FLAG and pCDH-LINC00116-FLAG contained the full-length of mouse 1500011K16Rik (447 bp, Supplementary Fig. 2a) and human LINC00116 transcript (442 bp, Supplementary Fig. 2b) with a FLAG tag, respectively; pCDH-MPM and pCDH-MPM-FS contained the coding sequence of mouse MPM (171 bp) and the full-length of 1500011K16Rik transcript (447 bp, Supplementary Fig. 2a) with 1-base pair deletion in the second codon of MPM, respectively; pCDH-PGC-1α that contained the coding sequence of mouse PGC-1α (NM_008904.2), pCDH-shMPM-1, and pCDH-shMPM-2 that expressed siRNAs targeting the 133–153-nt and 332–352-nt sequences of mouse MPM transcript; Mito-DsRed and ER-DsRed that expressed mitochondrial targeted-DsRed and endoplasmic reticulum targeted-DsRed, respectively. All oligonucleotide sequences used for cloning are listed in Supplementary Table 2.

### Rapid-amplification of cDNA ends (RACE)

To amplify the 3′-end of mouse and human MPM transcript, total RNA from muscle tissues of C57BL/6 J mice and Hela cells were subjected to reverse transcription with a 3′ RACE-adaptor primer, followed by nested PCR using gene-specific primers (Nest PCR-primer) and an adaptor primer (3′ RACE-adaptor primer). The 5′-end of the mouse and human MPM transcript was characterized using a 5′-Full RACE Kit (D315, TaKaRa, Kyoto, Japan). The sequences of the PCR-amplified 5′- and 3′-end fragments were analyzed by direct sequencing. All oligonucleotide sequences used for RACE are listed in Supplementary Table 2.

### Cell transfection

RNA oligoes were transfected using Lipofectamine RNAiMAX (Invitrogen, Carlsbad, CA, USA). A final concentration of 20 nM duplex was used. Plasmids, including pCDH-1500011K16Rik-FLAG, pCDH-LINC00116-FLAG, Mito-DsRed, and ER-DsRed, were transfected with Lipofectamine 3000 (Invitrogen). Plasmids, including pCDH-MPM, pCDH-MPM-FS, pCDH-shNC, pCDH-shMPM-1, pCDH-shMPM-2, and pCDH-PGC-1α, were transfected by electroporation using Super Electroporator NEPA21 (NEPA GENE Co. Ltd., Chiba, Japan) under the following conditions: poring pulse, 150 V for 7.5 ms; transfer pulse, 20 V for 50 ms.

### Immunofluorescence staining

Immunofluorescence staining assay was performed to examine the expression of MPM and MHC.

### MPM antibody production

A custom polyclonal antibody against the C-terminal region of MPM micropeptide was generated by GenScript (Nanjing, China). Rabbits were immunized with a synthetic peptide using CQDKLAATQKKLDLA peptide which conjugated keyhole limpet hemocyanin as a carrier. Sera were collected and affinity purified against the peptide immunogen.

### Isolation of mitochondria and characterization of MPM sublocalization

Mitochondria were isolated from C2C12 myoblasts using a mitochondria isolation kit (89874, Thermo Fisher Scientific, Waltham, MA, USA). In brief, 2 × 10^7^ cells were washed twice with precooling 1 × PBS and lysed in mitochondrial isolation buffer, followed by differential centrifugation.

To characterize the sublocalization of MPM, the mitochondria pellet was resuspended in isotonic buffer (10 mM MOPS-KOH pH 7.2, 250 mM sucrose, and 1 mM EDTA) without proteinase K (AS12456, Asegene, Guangzhou, China), isotonic or hypotonic buffer (10 mM MOPS-KOH pH 7.2, 1 mM EDTA) with 5 μg/mL proteinase K, or hypotonic buffer containing 1% Triton X-100 and 5 μg/mL proteinase K, incubated at 4 °C for 15 min, followed by addition of protease inhibitor cocktail (B14012, Bimake, Houston, TX, USA) and PMSF (ST505, Beyotime, Shanghai, China). The pellets were collected by centrifugation at 12,000 × *g*, 4 °C for 5 min, homogenized in lysis buffer containing protease inhibitor cocktail and then subjected to western blotting. TOMM20 (translocase of outer mitochondrial membrane 20), AK2 (adenylate kinase 2), and ND5 (NADH dehydrogenase subunit 5) were used as the controls for the outer mitochondrial membrane protein, the intermembrane space protein, and the inner mitochondrial membrane protein, respectively.

### Analysis of gene expression

The expression level of target genes was analyzed by real time quantitative RT-PCR (qPCR) or western blotting.

### Mouse studies

All mouse experiments were approved by the Institutional Animal Care and Use Committee at Sun Yat-sen University. All procedures for animal experiments were performed in accordance with the Guide for the Care and Use of Laboratory Animals (NIH publication Nos. 80-23, revised 1996) and according to the institutional ethical guidelines for animal experiments. All functional experiments were performed using 5- to 8-week-old male mice on a pure C57BL/6 J background. The MPM^*−/−*^ mouse was generated using CRISPR-cas9 system to disrupt the coding frame of MPM. A founder with 1-base pair insertion that created a premature stop codon after codon 5 was chosen for further analysis. The mouse tissues were collected and immediately frozen in liquid nitrogen and stored at −80 °C.

Mouse muscle injury and regeneration experiment was performed by injecting CTX (9012–91–3, BOYAO, Shanghai, China) into mouse gastrocnemius muscle. The size of myofibers was analyzed by hematoxylin–eosin (H&E) staining. Grip strength test, rotarod test, and weight-loaded swimming test were used to examine the muscle performance in MPM^+/+^ and MPM^−/−^ mice. The locomotion, pain response, and neurological reflexes of mice were evaluated by gait analysis, hot plate test, and unconditioned reflex tests. Oral temperature of 8-week-old male mice were recorded hourly from 9:00 a.m. to 4:00 p.m. by electronic thermometer (GENIAL TECHNOLOGY, Guangzhou, China) under room temperature (22 ± 1 °C).

### Measurement of oxygen consumption rate (OCR)

The OCR of C2C12 cells was examined by XF Cell Mito Stress Test (103015–100, Seahorse Bioscience, Billerica, MA, USA) using a Seahorse XF24 Extracellular Flux Analyzer (Seahorse Bioscience). Seahorse injection ports were loaded with a final concentration of 2 μM oligomycin (port A), 1 μM carbonyl cyanide 4-(trifluoromethoxy) phenylhydrazone (FCCP) (port B), and 0.5 μM rotenone and antimycin A (port C).

### Bioinformatics and statistical analysis

Mouse muscle transcriptome profile (SRA database: SRA001030), mouse Swiss-Prot protein database^50^, and microarray data (GEO accession number GSE4694) were used to identify myogenic differentiation-associated micropeptides

Data are expressed as the mean ± standard error of the mean (SEM) from at least three independent experiments. Unless otherwise noted, the differences between groups were analyzed using unpaired Student’s *t* test when only two groups were compared, and one-way ANOVA when more than two groups were compared. *P* < 0.05 was considered statistically significant. All statistical tests were two-sided and performed using GraphPad Prism (GraphPad Software Inc., San Diego, CA, USA).

## Supplementary information

supplementary information.

## References

[1] Andrews SJ, Rothnagel JA (2014). Emerging evidence for functional peptides encoded by short open reading frames. Nat. Rev. Genet..

[2] Huang JZ (2017). Apeptide encoded by a putative lncRNA HOXB-AS3 suppresses colon cancer growth. Mol. Cell..

[3] Guo B (2003). Humanin peptide suppresses apoptosis by interfering with Bax activation. Nature..

[4] Ikonen M (2003). Interaction between the alzheimer’s survival peptide humanin and insulin-like growth factor-binding protein 3 regulates cell survival and apoptosis. Proc. Natl Acad. Sci. USA.

[5] Kondo T (2010). Small peptides switch the transcriptional activity of Shavenbaby during Drosophila embryogenesis. Science..

[6] Pauli A (2014). Toddler: an embryonic signal that promotes cell movement via Apelin receptors. Science..

[7] Feige P, Brun CE, Ritso M, Rudnicki MA (2018). Orienting muscle stem cells for regeneration in homeostasis, aging, and disease. Cell Stem Cell..

[8] Jiang C (2014). Notch signaling deficiency underlies age-dependent depletion of satellite cells in muscular dystrophy. Dis. Model. Mech..

[9] Almada AE, Wagers AJ (2016). Molecular circuitry of stem cell fate in skeletal muscle regeneration, ageing, and disease. Nat. Rev. Mol. Cell Biol..

[10] Heslop L, Morgan JE, Partridge TA (2000). Evidence for a myogenic stem cell that is exhausted in dystrophic muscle. J. Cell Sci..

[11] Zammit PS (2006). Pax7 and myogenic progression in skeletal muscle satellite cells. J. Cell. Sci..

[12] Charge SB, Rudnicki MA (2004). Cellular and molecular regulation of muscle regeneration. Physiol. Rev..

[13] Crist CG, Montarras D, Buckingham M (2012). Muscle satellite cells are primed for myogenesis but maintain quiescence with sequestration of Myf5 mRNA targeted by microRNA-31 in mRNP granules. Cell Stem Cell.

[14] Yu X (2017). Long non-coding RNA Linc-RAM enhances myogenic differentiation by interacting with MyoD. Nat. Commun..

[15] Matsumoto A (2017). mTORC1 and muscle regeneration are regulated by the LINC00961-encoded SPAR polypeptide. Nature..

[16] Bi P (2018). Fusogenic micropeptide myomixer is essential for satellite cell fusion and muscle regeneration. Proc. Natl Acad. Sci. USA.

[17] Shi J (2017). Requirement of the fusogenic micropeptide myomixer for muscle formation in zebrafish. Proc. Natl Acad. Sci. USA.

[18] Bi P (2017). Control of muscle formation by the fusogenic micropeptide myomixer. Science..

[19] Zhang Q (2017). The microprotein minion controls cell fusion and muscle formation. Nat. Commun..

[20] Wu Z (1999). Mechanisms controlling mitochondrial biogenesis and respiration through the thermogenic coactivator PGC-1. Cell..

[21] Choi J (1990). MyoD converts primary dermal fibroblasts, chondroblasts, smooth muscle, and retinal pigmented epithelial cells into striated mononucleated myoblasts and multinucleated myotubes. Proc. Natl Acad. Sci. USA.

[22] Weintraub H (1991). The myoD gene family: nodal point during specification of the muscle cell lineage. Science..

[23] Olson EN (1992). Interplay between proliferation and differentiation within the myogenic lineage. Dev. Biol..

[24] Chen JF (2006). The role of microRNA-1 and microRNA-133 in skeletal muscle proliferation and differentiation. Nat. Genet..

[25] Qiu H (2016). MicroRNA-17-92 regulates myoblast proliferation and differentiation by targeting the ENH1/Id1 signaling axis. Cell. Death. Differ..

[26] Legnini I, Morlando M, Mangiavacchi A, Fatica A, Bozzoni I (2014). A feedforward regulatory loop between HuR and the long noncoding RNA linc-MD1 controls early phases of myogenesis. Mol. Cell..

[27] Gong C (2015). A long non-coding RNA, LncMyoD, regulates skeletal muscle differentiation by blocking IMP2-mediated mRNA translation. Dev. Cell..

[28] Tieland M, Trouwborst I, Clark BC (2018). Skeletal muscle performance and ageing. J. Cachexia Sarcopenia Muscle..

[29] Lexell J, Henriksson-Larsen K, Winblad B, Sjostrom M (1983). Distribution of different fiber types in human skeletal muscles: effects of aging studied in whole muscle cross sections. Muscle Nerve..

[30] Rogers MA, Hagberg JM, Martin WH, Ehsani AA, Holloszy JO (1990). Decline in VO2max with aging in master athletes and sedentary men. J. Appl. Physiol..

[31] Short KR (2005). Decline in skeletal muscle mitochondrial function with aging in humans. Proc. Natl Acad. Sci. USA.

[32] Proctor DN, Joyner MJ (1997). Skeletal muscle mass and the reduction of VO2max in trained older subjects. J. Appl. Physiol..

[33] Broskey NT (2014). Skeletal muscle mitochondria in the elderly: effects of physical fitness and exercise training. J. Clin. Endocrinol. Metab..

[34] Lanza IR, Nair KS (2009). Muscle mitochondrial changes with aging and exercise. Am. J. Clin. Nutr..

[35] McBride HM, Neuspiel M, Wasiak S (2006). Mitochondria: more than just a powerhouse. Curr. Biol..

[36] Wagatsuma A, Sakuma K (2013). Mitochondria as a potential regulator of myogenesis. Sci. World J..

[37] Sin J (2016). Mitophagy is required for mitochondrial biogenesis and myogenic differentiation of C2C12 myoblasts. Autophagy..

[38] Esteban-Martinez L (2017). Programmed mitophagy is essential for the glycolytic switch during cell differentiation. EMBO J.

[39] Mansueto G (2017). Transcription factor EB controls metabolic flexibility during exercise. Cell Metab..

[40] Tezze C (2017). Age-associated Loss of OPA1 in muscle impacts muscle mass, metabolic homeostasis, systemic inflammation, and epithelial senescence. Cell Metab..

[41] Friis RM (2014). Rewiring AMPK and mitochondrial retrograde signaling for metabolic control of aging and histone acetylation in respiratory-defective cells. Cell Rep.

[42] Monaghan RM (2015). A nuclear role for the respiratory enzyme CLK-1 in regulating mitochondrial stress responses and longevity. Nat. Cell Biol..

[43] Luo Y, Bond JD, Ingram VM (1997). Compromised mitochondrial function leads to increased cytosolic calcium and to activation of MAP kinases. Proc. Natl Acad. Sci. USA.

[44] Mouchiroud L (2013). The NAD(+)/sirtuin pathway modulates longevity through activation of mitochondrial UPR and FOXO signaling. Cell..

[45] Kim JH (2018). Mitochondrial ROS-derived PTEN oxidation activates PI3K pathway for mTOR-induced myogenic autophagy. Cell Death Differ..

[46] van Wessel T, de Haan A, van der Laarse WJ, Jaspers RT (2010). The muscle fiber type-fiber size paradox: hypertrophy or oxidative metabolism?. Eur. J. Appl. Physiol..

[47] Overmyer KA (2015). Maximal oxidative capacity during exercise is associated with skeletal muscle fuel selection and dynamic changes in mitochondrial protein acetylation. Cell Metab..

[48] Stein CS (2018). Mitoregulin: a lncRNA-encoded microprotein that supports mitochondrial supercomplexes and respiratory efficiency. Cell Rep.

[49] Makarewich CA (2018). MOXI Is a mitochondrial micropeptide that enhances fatty acid beta-oxidation. Cell Rep..

[50] UniProt C (2013). Update on activities at the Universal Protein Resource (UniProt) in 2013. Nucleic Acids Res..

