# Supplementary material for: A novel mitochondrial micropeptide MPM enhances mitochondrial respiratory activity and promotes myogenic differentiation
Source: Cell Death Dis. 2019 Jul 11;10(7):528. doi: 10.1038/s41419-019-1767-y (PMC6624212; doi:10.1038/s41419-019-1767-y)
Supplement: Supplementary file 1 — supplementary information. [file 41419_2019_1767_MOESM1_ESM.doc]

**A novel mitochondrial micropeptide MPM enhances mitochondrial respiratory activity and promotes myogenic differentiation**

Yi-Fang Lin, Man-Huan Xiao, Hua-Xing Chen, Yu Meng, Na Zhao, Liang Yang, Haite Tang, Jia-Lei Wang, Xingguo Liu, Ying Zhu and Shi-Mei Zhuang

**Inventory of supplementary data**

1. Supplementary Materials and Methods...………. .….…. . . .. . . Page 2 - 7

2. Supplementary Figures and legends……….….. .. .. .. ...……… Page 8 - 17

3. Supplementary Tables...………….…....…….….…….. ..….…. Page 18 - 21

**Supplementary Materials and Methods**

***Vector construction***

The following plasmids were used: pCDH-1500011K16Rik-FLAG, pCDH-LINC00116-FLAG, pCDH-MPM, pCDH-MPM-FS, pCDH-PGC-1α, pCDH-shNC, pCDH-shMPM-1, pCDH-shMPM-2, Mito-DsRed and ER-DsRed.

To create pCDH-1500011K16Rik-FLAG and pCDH-LINC00116-FLAG, the full-length of mouse short isoform of 1500011K16Rik (447 bp, Supplementary Fig. 2a) or human LINC00116 (442 bp) with a FLAG epitope tag before its stop codon was amplified by fusion PCR and inserted into the *Eco*RI*/Bam*HI sites of pCDH-CMV-MCS-EF1-Puro vector (System Biosciences, Palo Alto, CA, USA). To create pCDH-MPM and pCDH-MPM-FS, the coding sequence of mouse MPM or the full-length of mouse short isoform of *1500011K16Rik* with 1-base pair deletion in the second codon of MPM was inserted into the *Eco*RI/*Bam*HI sites of pCDH-CMV-MCS-EF1-Puro vector (System Biosciences). To construct pCDH-PGC-1α vector, the coding sequence of mouse PGC-1α (NM_008904.2) was inserted into the *Swa*I/*Bam*HI sites of pCDH-CMV-MCS-EF1-Puro vector. To construct pCDH-shNC and pCDH-shMPM vectors, the fragments containing the U6 promoter plus shRNA sequences were generated by PCR and inserted between the *Spe*I and *Bam*HI sites in pCDH-CMV-MCS-EF1-Puro vector, resulting in a replacement of CMV promoter to U6 promoter. Mito-DsRed and ER-DsRed were gifts from Prof. György Hajnóczky (MitoCare Center, Thomas Jefferson University), and were subcloned into pRlenti^1^ and pcDNA3.0 vectors (Invitrogen, Carlsbad, CA, USA), respectively.

All constructs were verified by direct [DNA sequencing](https://www.sciencedirect.com/topics/biochemistry-genetics-and-molecular-biology/dna-sequencing). All oligonucleotide sequences used for cloning are listed in Supplementary Table 2.

***Immunofluorescence staining***

Cells were fixed with 4% paraformaldehyde for 15 min, permeabilized with 0.25% Triton X-100, blocked with blocking buffer (1% bovine serum albumen in PBS) for 30 minutes, stained by rabbit monoclonal antibody (mAb) against FLAG (14793, Cell Signaling Technology, CST, Beverly, MA, USA) at 4 °C overnight or mouse mAb against MHC (MF20; 53-6503-82, Thermo Fisher Scientific, Waltham, MA, USA) at room temperature for 2 hours, followed by incubation with or without goat anti-rabbit Alexa Fluor 488 secondary antibody (A-11008, Thermo Fisher Scientific) at room temperature for 1 hour, and then counterstaining with 4’, 6’-diamidino-2-phenylindole (DAPI; Sigma-Aldrich, St. Louis, MO, USA). For MPM-FLAG staining, samples were imaged using a Zeiss LSM-800 confocal with a 63 × oil objective (Oberkochen, Germany). For MHC staining, fluorescent pictures were photographed with Nikon ECLIPSE TI fluorescence microscope.

***Analysis of gene expression***

Real-time quantitative polymerase chain reaction (qPCR) assays were performed to evaluate the RNA levels. Total RNA was extracted using TRIzol reagent (Invitrogen) and reverse-transcribed using M-MLV reverse transcriptase (M1701, Promega, Madison, WI, USA). qPCR was performed on a LightCycler 480 (Roche Diagnostics, Germany, Mannheim, Germany) using 2 × SYBR Green qPCR Master Mix (B21202, Bimake, Houston, TX, USA). All reactions were performed in duplicate. The cycle threshold (Ct) values differed by less than 0.5 between duplicate wells. The relative expression levels of the target genes were normalized to that of internal control genes, which yielded a 2^-ΔCt^ value. U6 was used as the internal control gene. Sequences for primers are listed in Supplementary Table 2.

Western blotting was used to examine protein levels. Total cell or tissue lysates were resolved in Tricine-SDS-polyacrylamide gels^2^, electrophoretically transferred to polyvinylidene difluoride membranes (Millipore, Billerica, MA, USA), and then detected with antibodies. The antibodies used included: rabbit polyclonal antibodies against MPM (GenScript, Nanjing, China), GAPDH (BA2913, Boster, Wuhan, China), VDAC1 (BA3754, Boster), TOMM20 (ab78547, abcam, Cambridge, MA, USA), AK2 (D221850, BBI, Shanghai, China), ND5 (A8135，Abclonal, Wuhan, China) and COX4 (AC610, Beyotime, Shanghai, China); rabbit monoclonal antibody (mAb) against FLAG (14793, CST); mouse mAb against Histone 3 (AF0009, Beyotime).

***Mouse studies***

To generate MPM*^-/-^* mouse, CRISPR-cas9 system was employed to disrupt the coding frame of MPM*.* The pT7-3XFlag-hCas9 plasmid was linearized with PmeI to synthesize Cas9 mRNA using the mMACHINE T7 ULTRA kit (AMB13455, Life Technologies). The pDR274 vector encoding gRNA sequences was *in vitro* transcribed using the MEGAshortscript T7 kit (AM1354, Life Technologies). The Cas9 mRNA and the gRNA were subsequently purified with the MEGAclear kit (AM1908, Life Technologies), re-suspended in RNase-free water, and quantified using NanoDrop-1000 (Nanodrop,Wilmington, DE, USA). The mixture of Cas9 mRNA and gRNA was injected into 0.5-day zygotes of C57BL/6J mice. The injected zygotes were transplanted into the oviduct of 0.5-day pseudo-pregnant mothers 3 hours after injection. CRISPR/Cas9 KO mice were bred with WT mice for more than three generations to avoid off-target effects. Mouse genotyping was done by PCR and sequencing of tail-snips by Mouse Genotyping Kit (KK7302, KAPA Biosystems, Boston, MA, USA) following the manufacturer’s instructions.

For mouse muscle injury and regeneration experiment, five-week-old male mice were anaesthetized with 1% pelltobarbitalum natricum (40 mg/kg of body mass, [57-33-0](https://www.chemicalbook.com/CAS_57-33-0.htm), LAN TAI CHEM, Beijing, China), followed by injection of 50 µL 20 μM CTX in 0.9% NaCl solution (9012-91-3, BOYAO, Shanghai, China) or 50 µL 0.9% NaCl. The injected muscles were collected at day 1, 3, 5 and 7 post-injection, fixed in 4% paraformaldehyde for hematoxylin-eosin staining or frozen in liquid nitrogen for RNA and protein extraction.

The maximal grip force of limb was detected in eight-week-old male mice using grip force meter (Columbus Instruments, Columbus, Ohio). Mice were placed on a wire grid with forelimbs and hind limbs gripping the grid, pulled backward by the tail, and the maximal force that animal exerted at the time of release was recorded. Five trials were carried out consecutively and the maximum value of the combined forelimb/hindlimb grip strength represented maximum grip force of mice.

For rotarod test, eight-week-old male mice were subjected to forced exercise on a Rotarod system (Columbus Inst). Mice were trained on the accelerating rod (0-20 rpm) for 30 mintutes for 2 consecutive days. On the test day, the mice were acclimated to the rotarod test for 10 minutes. After two hours resting, they were placed on the rotating cylinder with constant speed (32 rpm). The length of time that each animal was able to stay on the rotarod was recorded as the latency to fall^3^.

For weight-loaded swimming test, swimming exercise was conducted with mice carrying a load of 10% of their body weight. Swimming exercise was carried out in a tank with 35-cm deep, 25 ± 1 °C water. Eight-week-old male mice were trained to swim without load for 10 min twice per week for two weeks. On the test day, the swiming time before exhaustion was recorded. The exhaustion was defined as the mouse failed to rise to the surface of the water to breathe within 7 seconds^4^.

For gait analysis, mice were manually restrained and their hindpaws dipped in nontoxic ink. Animals were allowed to walk on white paper within a restricted area (70 × 400 mm). Stride length (the distance between consecutive left or right paw prints), base length (the distance perpendicular to parallel left and right paw prints), and overlap length were measured^5^.

For hot plate test, mice were placed on a metallic plate heated at 55 °C within an acrylic container. Latency to response, such as lifting or licking a hind paw, jumping, or vocalization, was recorded. Three measurements were performed at a minimum of 5-minute intervals for each mouse and the averaged latency was achieved^6^.

To check the nerological reflexes, the righting reflex, postural reflex, eye blink reflex, whisker-orienting reflex, and pupil constriction and dilation reflex were evaluated. In righting reflex test, mice were turned on its back, and the time to right itself to an upright position was measured^7^. In postural reflex, mice were put in a small cage and shaken up and down and left and right. Existence of an appropriate response (animals splaying their four feet) was scored^7^. In eye blink reflex test, the paradigms were measured by touching the eye with fibers tight from a cotton-tip swab^8^. In whisker-orienting reflex test, the whiskers of a freely moving mouse were touched lightly, and the response of mouse was recorded when the continuous movements of the whiskers stopped^8^. In pupil constriction and dilation reflex test, a penlight or a small flashlight beam was directed at the eye of the mice in a dimly lit room, and the constriction and following dilation of the pupils were observed when the light was removed^8^.

***Hematoxylin-eosin staining***

Formalin-fixed, paraffin-embedded tissues were cut into 5-μm sections, deparaffinized in xylene, rehydrated through graded ethanol. Sections were subjected to hematoxylin (Jiu Zhou Bai Lin, Beijing, China) staining for 5 minutes and eosin (ZSGB-BIO, Beijing, China) staining for 3 minutes.

***Bioinformatic analysis***

To identify myogenic differentiation-associated micropeptides, RNA-seq data of mouse muscle transcriptome profile was obtained from the SRA database (mouse: SRA001030). The overlapping transcripts from the SRA database and the Swiss-Prot protein database (version57.2; 16,115 entries) were used to identify the transcripts with micropeptide-coding potential. Differential expressed genes during differentiation of C2C12 myoblasts into myotubes were derived from microarray data (GEO accession number GSE4694) based on the GPL1261 platform (Affymetrix Mouse Genome 430 2.0 Array; Affymetrix; Thermo Fisher Scientific, Inc, Waltham, MA, USA).

**Supplementary references**

1. Bao, F. X. et al. Mitochondrial Membrane Potential-dependent Endoplasmic Reticulum Fragmentation is an Important Step in Neuritic Degeneration. *CNS Neurosci. Ther.* **22**, 648-660, (2016).

2. Schagger, H. Tricine-SDS-PAGE. *Nat. Protoc.* **1**, 16-22, (2006).

3. Rozas, G. & Labandeira Garcia, J. L. Drug-free evaluation of rat models of parkinsonism and nigral grafts using a new automated rotarod test. *Brain Res.* **749**, 188-199, (1997).

4. Yang, Q. et al. Effects of macamides on endurance capacity and anti-fatigue property in prolonged swimming mice. *Pharm. Biol.* **54**, 827-834, (2016).

5. Hamill, R. W. et al. Autonomic dysfunction and plasticity in micturition reflexes in human alpha-synuclein mice. *Dev. Neurobiol.* **72**, 918-936, (2012).

6. Mogil, J. S. et al. Heritability of nociception I: responses of 11 inbred mouse strains on 12 measures of nociception. *Pain* **80**, 67-82, (1999).

7. Santos, M., Silva-Fernandes, A., Oliveira, P., Sousa, N. & Maciel, P. Evidence for abnormal early development in a mouse model of Rett syndrome. *Genes Brain Behav.* **6**, 277-286, (2007).

8. Anegon, I. *Rat Genomics: Methods and Protocols Ch. 24* (Humana Press, New York, 2010).

**Supplementary Figures and legends**

**
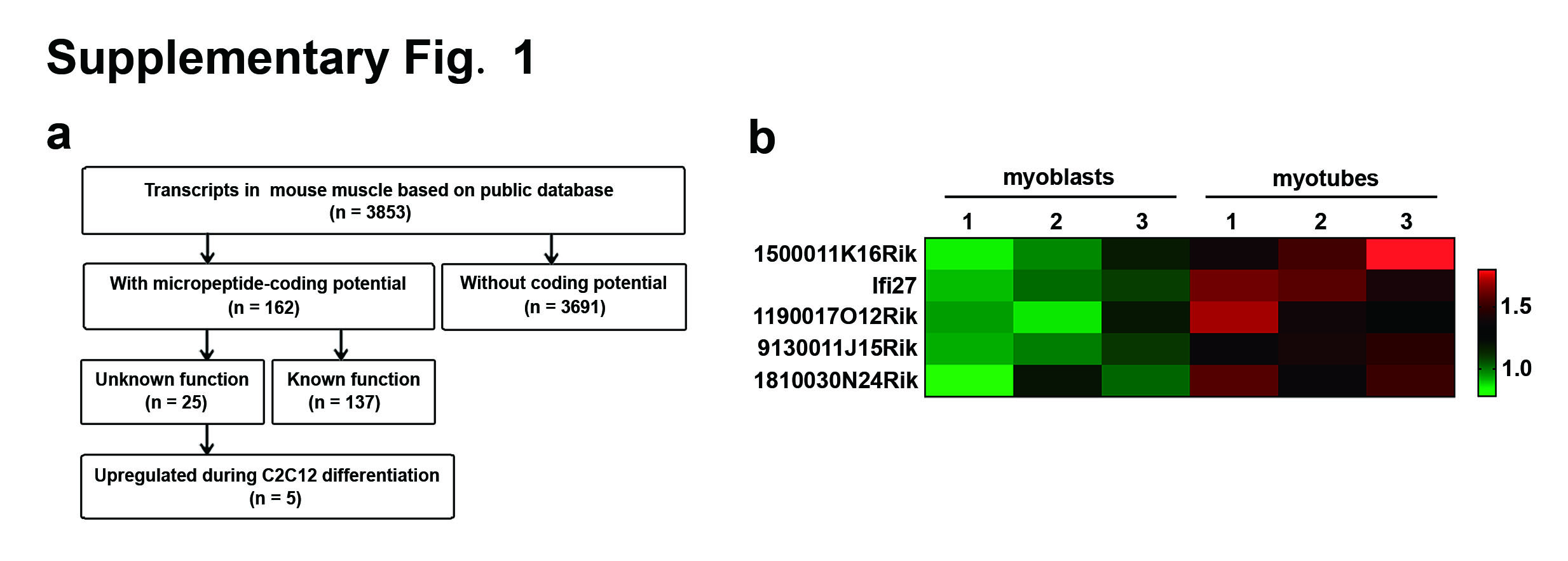
**

**Supplementary Fig. 1 The screening for novel micropeptides associated with myogenic differentiation. a** The screening workflow for candidate micropeptide transcripts. **b** Heatmap representing the expression of 5 candidate micropeptide transcripts in myoblasts and myotubes from microarray data (GSE4694).

**
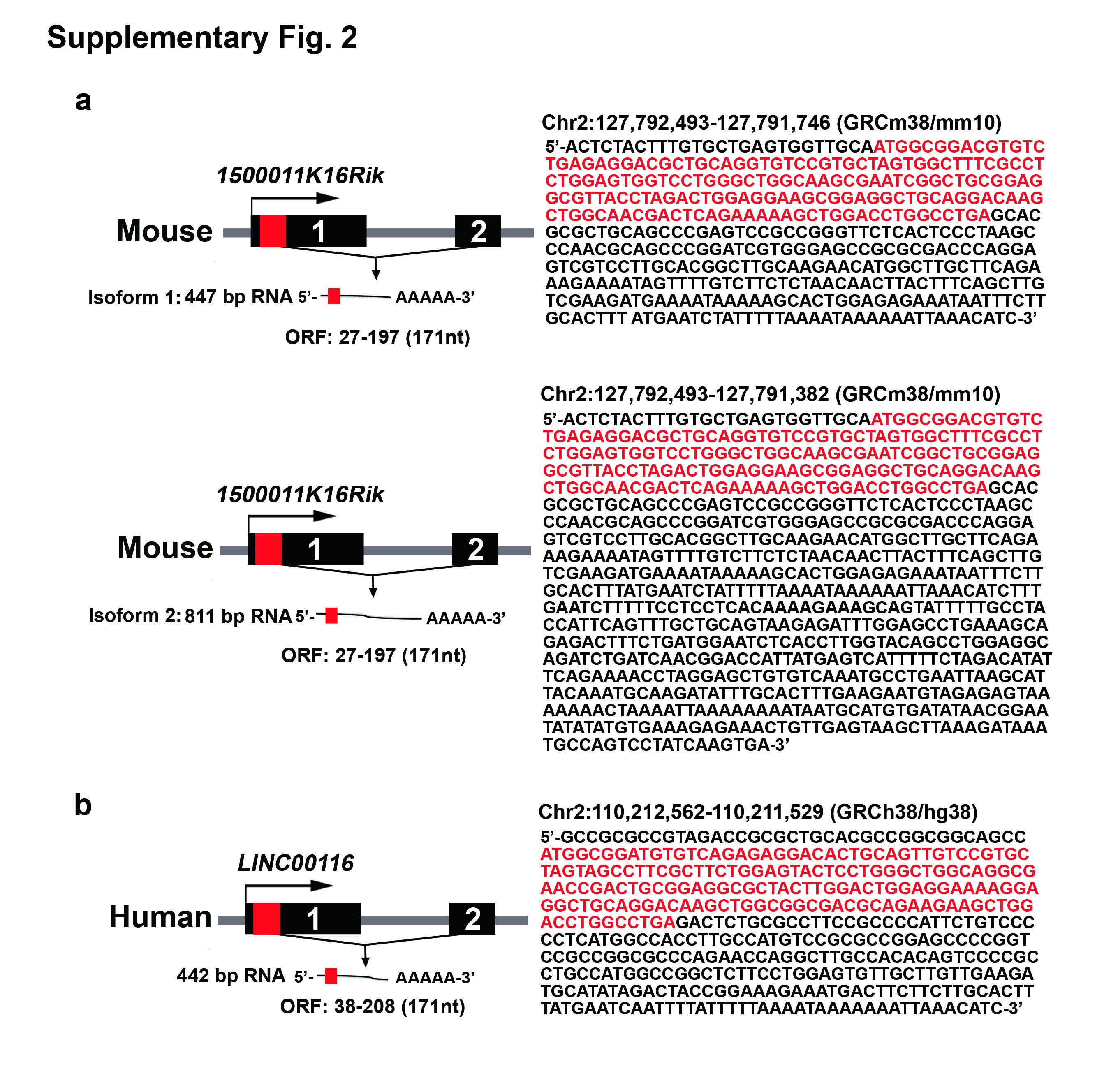
**

**Supplementary Fig. 2 Schematic diagram and nucleotide sequences of** **1500011K16Rik and LINC00116 transcripts.** The sORF within exon 1 of mouse 1500011K16Rik (a) or human LINC00116 (b) transcripts are depicted as red rectangles in *left* *panel*. Nucleotide sequences of the sORFs are shown in red in *right panel*.

**
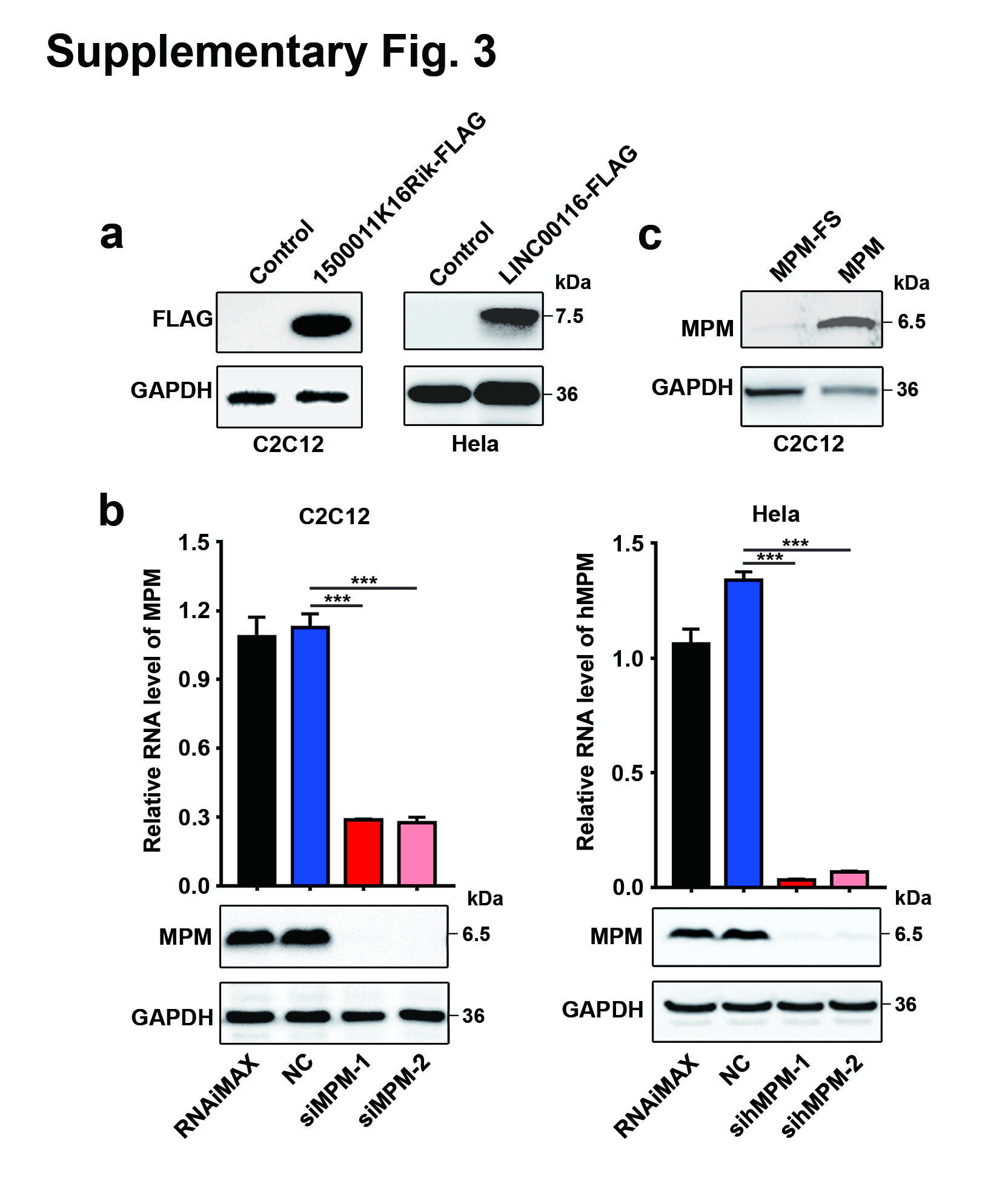
**

**Supplementary Fig. 3 The 1500011K16Rik/LINC00116 transcript encodes a ~6.5 kDa micropeptide. a** Detection of exogenous fusion protein with anti-FLAG antibody. C2C12 and Hela cells were transfected with the indicated plasmids for 48 hours before Western blotting. **b** Detection of endogenous MPM protein with anti-MPM antibody. C2C12 and Hela cells were transfected with the indicated RNA duplexes for 48 hours and then subjected to qPCR (*upper*) or Western blotting (*lower*). **c** Detection of ectopic expression of MPM with anti-MPM antibody. C2C12 myoblasts were transfected with pCDH-MPM-FS or pCDH-MPM plasmids for 48 hours before western blotting. Data are presented as mean ± SEM in (b); ***, *P* < 0.001.


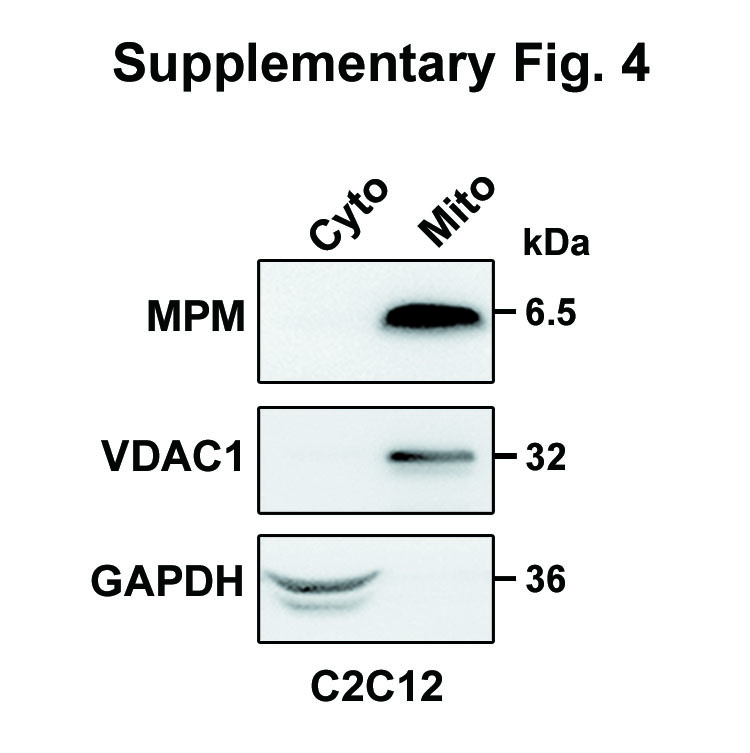


**Supplementary Fig. 4 MPM is localized in mitochondria.** Immunoblotting of endogenous MPM, VDAC1 and GAPDH in the mitochondrial and cytosol fractions of C2C12 myoblasts.

**
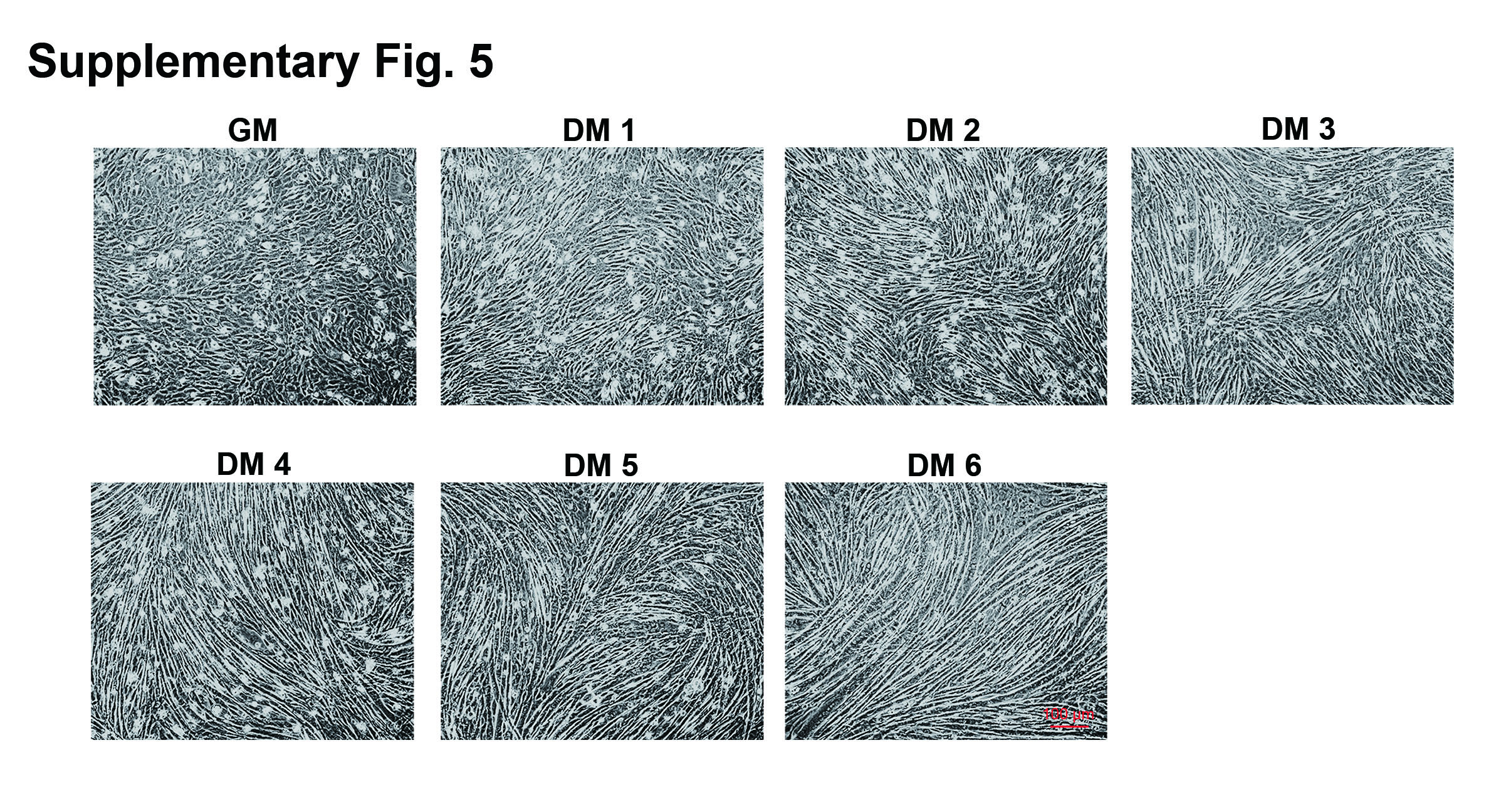
**

**Supplementary Fig. 5** **Phase-contrast images of the differentiating C2C12 cells.** C2C12 myoblasts were cultured in 10% FBS-containing DMEM (GM) to reach 90% confluence, then incubated in differentiation medium (DM) for 1-6 days. Scale bar, 100 μm.

**
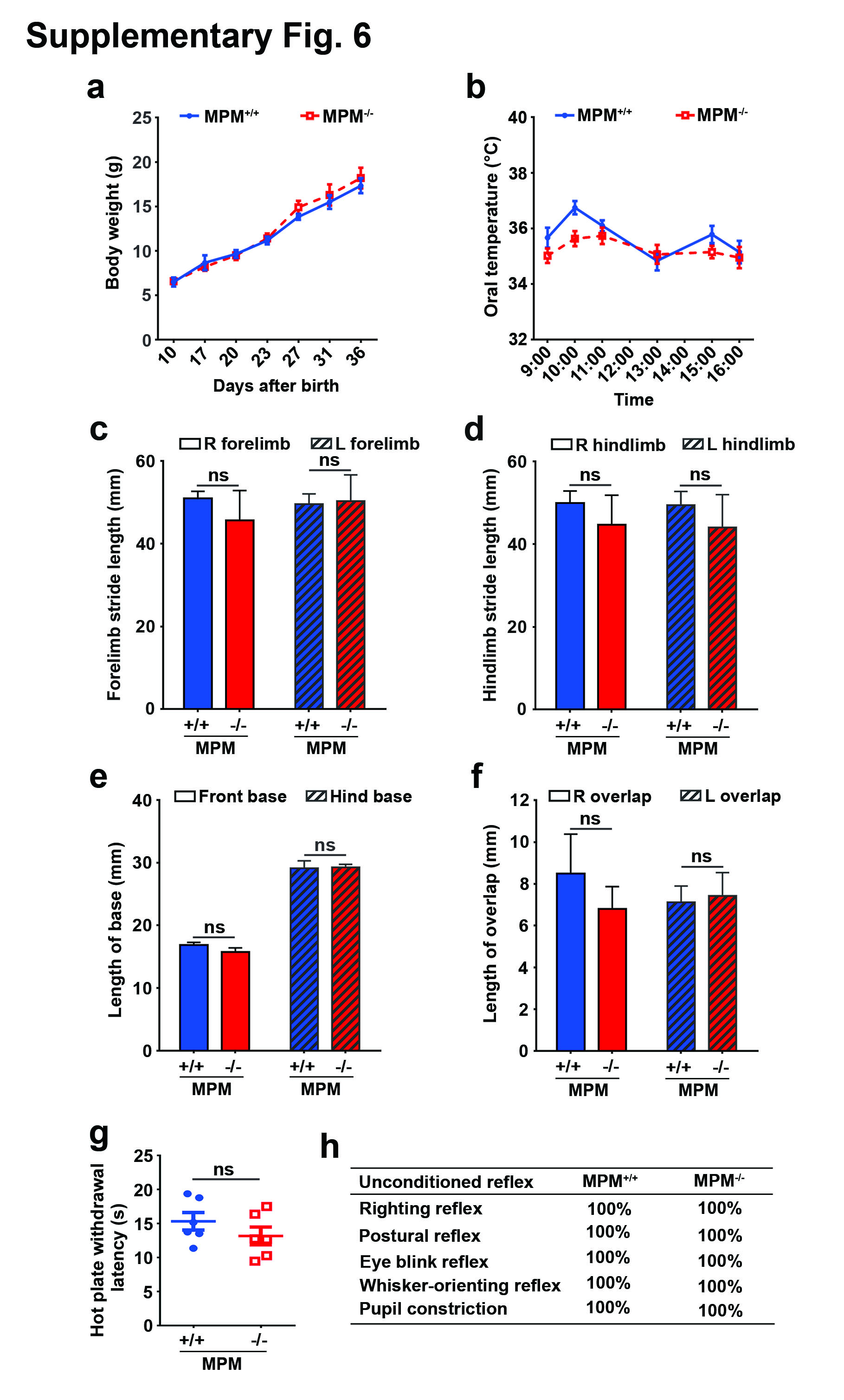
**

**Supplementary Fig. 6 Examination on the body weight, oral temperature, gait, sensitivity of pain and unconditioned reflexes of MPM^+/+^ and MPM^-/-^ mice.** **a** There was no difference in body weight between MPM^+/+^ mice and MPM^-/-^ mice. The body weight was recorded at the indicated time for MPM^+/+^ (n = 3) and MPM^-/-^ (n = 5) male mice. **b** Knockout of MPM did not affect mouse oral temperature. Oral temperature was recorded every hour from 9:00 am to 4:00 pm in MPM^+/+^ (n = 6) and MPM^-/-^ (n = 6) mice. **c-f** MPM^-/-^ mice exhibited normal gaits. The stride length (c, d), base length (e) and right/left overlap length (f) of limbs were measured in MPM^+/+^ mice (n = 4) and MPM^-/-^ mice (n = 3). **g** Knockout of MPM did not affect mouse sensitivity of pain. Hot plate test was applied to MPM^+/+^ (n = 6) and MPM^-/-^ (n = 6) mice. **h** Knockout of MPM did not affect the unconditioned reflex. The unconditioned reflexes were detected in MPM^+/+^ (n = 5) and MPM^-/-^ (n = 5) mice. The number in the chart represented the percentage of mice which reacted normally. The six to eight-week-old male mice were used in (b-h). Data are presented as mean ± SEM in (c-f). ns, not significant.

**
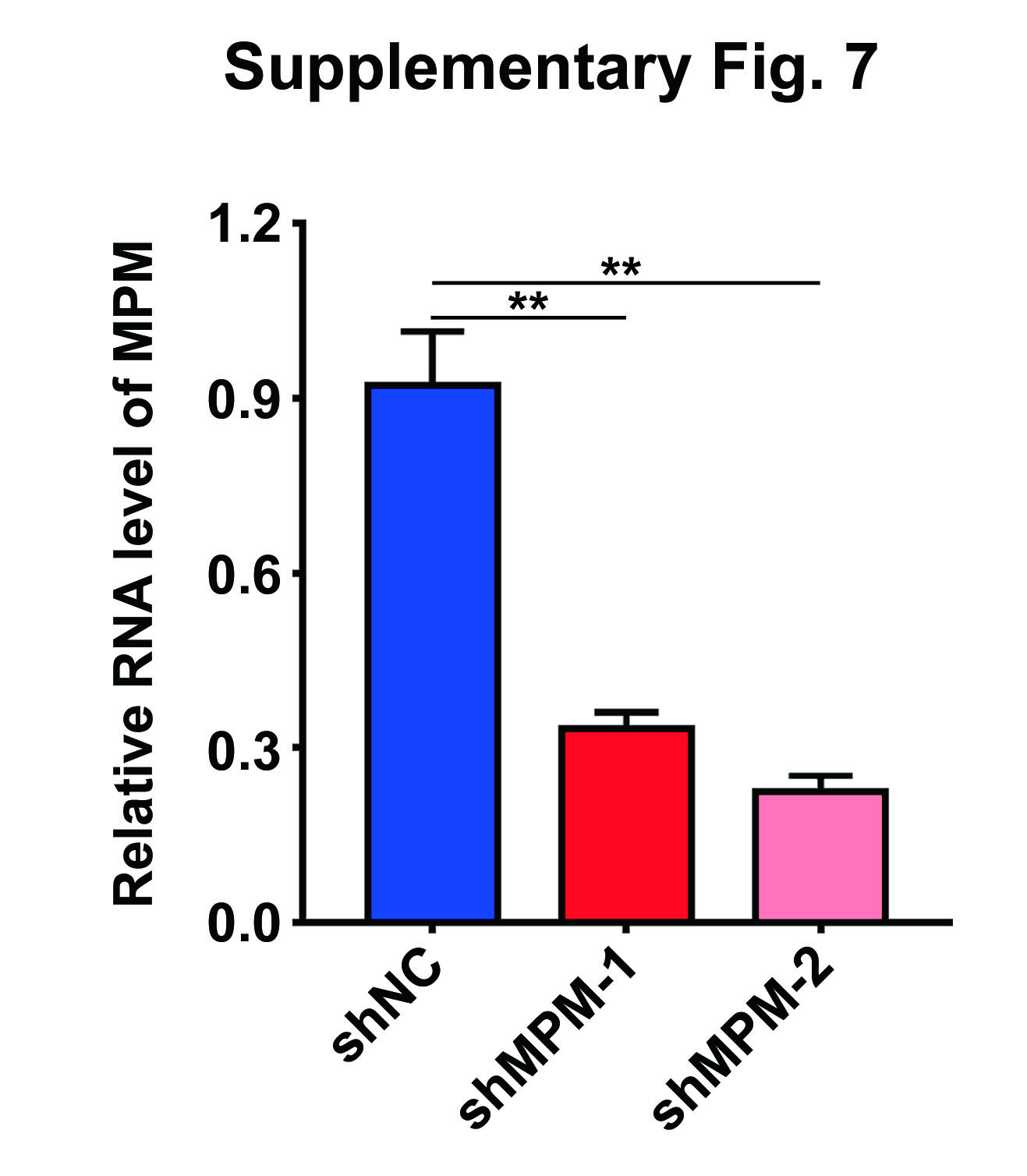
**

**Supplementary Fig. 7 The knockdown efficiency of shMPM-1 and shMPM-2 plasmids.** C2C12 myoblasts were transfected with the indicated plasmids for 48 hours before qPCR assay. Data are presented as mean ± SEM; **, *P* < 0.01.

**
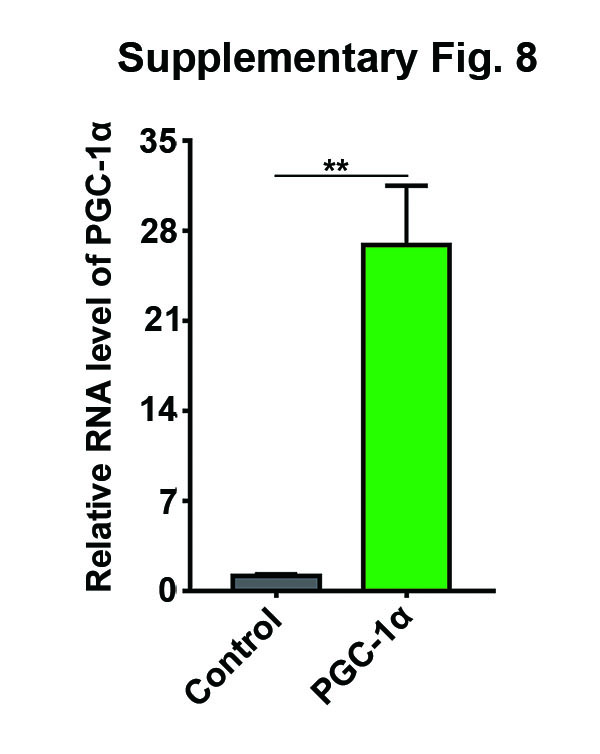
**

**Supplementary Fig. 8 The overexpression efficiency of PGC-1α** **plasmids.** C2C12 myoblasts were transfected with the indicated plasmids for 84 hours, then subjected to qPCR assay for PGC**-**1α level. Data are presented as mean ± SEM; **, *P* < 0.01.

**Supplementary Tables**

**Supplementary Table 1. Twenty-five muscle-related transcripts with micropeptide-coding potential^a^**

| **Gene ID** | **Gene Symbol** | **RPKM^b^** |
| --- | --- | --- |
| 70257 | 2010107E04Rik | 631.23 |
| 67885^c^ | 1500011K16Rik | 487.83 |
| 235973 | A630095E13Rik | 226.3 |
| 26901 | Deb1 | 92.7 |
| 66119 | 1110002E23Rik | 77.95 |
| 76933 | Ifi27 | 73.2 |
| 68936 | 1190017O12Rik | 62.94 |
| 73737 | 1110008P14Rik | 38.05 |
| 66433 | Chchd7 | 36.49 |
| 67248 | Rpl39 | 31.14 |
| 80284 | BC003266 | 31.08 |
| 66177 | Ubl5 | 30.37 |
| 58239 | Dexi | 30.33 |
| 59095 | Fxyd6 | 20.9 |
| 66818 | 9130011J15Rik | 17.62 |
| 84704 | Snurf | 17.34 |
| 66425 | Pcp4l1 | 16.07 |
| 66291 | 1810030N24Rik | 15.92 |
| 68512 | 1110019J04Rik | 13.84 |
| 69161 | Manbal | 13.67 |
| 71683 | Gypc | 13.57 |
| 68552 | 1110003E01Rik | 13.34 |
| 170748 | BC017612 | 11.8 |
| 108934 | BC024659 | 11.7 |

^a^Mouse muscle transcriptome profile from SRA001030.

^b^RPKM, Reads Per Kilobase per Million mapped reads, data from SRA001030.

^c^Transcripts upregulated during differentiation of C2C12 myoblasts into myotubes are indicated in red, based on GSE4694.

**Supplementary Table 2. Sequences of RNA and DNA oligonucleotides**

| **Name** | **Sense strand/Sense primer (5'-3')** | **Antisense strand/Antisense primer (5'-3')** |
| --- | --- | --- |
| **siRNA duplexes** |  |  |
| NC | UUGUACUACACAAAAGUACUG | GUACUUUUGUGUAGUACAGUU |
| siMPM-1 | GGACAAGCUGGCAACGACUdTdT | AGUCGUUGCCAGCUUGUCCdTdG |
| siMPM-2 | CUUUCAGCUUGUCGAAGAUdTdT | AUCUUCGACAAGCUGAAAGdTdA |
| sihMPM-1 | AGCCUUCGCUUCUGGAGUAdTdT | UACUCCAGAAGCGAAGGCUdAdC |
| sihMPM-2 | GCAUAUAGACUACCGGAAAdTdT | UUUCCGGUAGUCUAUAUGCdAdT |
| **Primers for cloning (restriction enzyme sites are underlined)** | |  |
| pCDH-1500011K16Rik-FLAG  fusion PCR outer primer | CGAGAATTCGCGCCAATCCGTAGCTCA | ACGGGATCCGATGTTTAATTTTTTATTTTAAAAATAG |
| pCDH-1500011K16Rik-FLAG  fusion PCR inner primer | GCCGATTACAAGGATGACGATGACAAGTGAGCACGCGCTGCAGCCC | TCACTTGTCATCGTCATCCTTGTAATCGGCCAGGTCCAGCTTTTTC |
| pCDH-LINC00116-FLAG  fusion PCR outer primer | CGAGAATTCGCCGCGCCGTAGACCG | ACGGGATCCGATGTTTAATTTTTTTATTT |
| pCDH-LINC00116-FLAG  fusion PCR inner primer | GCCGATTACAAGGATGACGATGACAAGTGAGACTCTGCGCCTTCCG | TCACTTGTCATCGTCATCCTTGTAATCGGCCAGGTCCAGCTTCTTCT |
| pCDH-MPM | CGAGAATTCATGGCGGACGTGTCTGAGAG | ACGGGATCCTCAGGCCAGGTCCAGCTTTT |
| pCDH-MPM-FS | CGAGAATTCGCGCCAATCCGTAGCTCACTCTACTTTGTGCTGAGTGGTTGCAATGCGGACGTGTC | ACGGGATCCGATGTTTAATTTTTTATTTTAAAAATAG |

**Supplementary Table 2. Sequences of RNA and DNA oligonucleotides (Continued)**

| **Name** | **Sense strand/Sense primer (5'-3')** | **Antisense strand/Antisense primer (5'-3')** |
| --- | --- | --- |
| shMPM-1 | CATACTAGTTCGGGCAGGAAGAGGGCCTA | CATGGATCCAAAAAGGACAAGCTGGCAACGACTTCTCTTGAAAGTCGTTGCCAGCTTGTCCGAATTCCGCGTCCTTTCCACAAG |
| shMPM-2 | CATACTAGTTCGGGCAGGAAGAGGGCCTA | CATGGATCCAAAAACTTTCAGCTTGTCGAAGATTCTCTTGAAATCTTCGACAAGCTGAAAGCGAATTCCGCGTCCTTTCCACAAG |
| pCDH-PGC-1α | CGCATTTAAATATGGCTTGGGACATGTGCAGCCAAGACT | CGCGGATCCTTACCTGCGCAAGCTTCTCTGAGCTT |
| **Primers for genotyping of MPM^+/+^ and MPM^-/-^ mice** | | |
| MPM genomic DNA | TGTAGAAGAAACGCGGAGCC | GCTTAGGGAGTGAGAACCCG |
| **Primers for qPCR** | | |
| GAPDH (mouse) | AACTTTGGCATTGTGGAAGG | CACATTGGGGGTAGGAACAC |
| MPM (mouse) | GTGTCCGTGCTAGTGGCTTT | CCAGGTCCAGCTTTTTCTGA |
| MPM (human) | TGTGTCAGAGAGGACACTGC | CCTTTTCCTCCAGTCCAAGT |
| MHC (mouse) | CGCAAGAATGTTCTCAGGCT | GCCAGGTTGACATTGGATTG |
| U6 | CTCGCTTCGGCAGCACA | AACGCTTCACGAATTTGCGT |
| Pax7 (mouse) | AGGCAGCAAGCCCAGACAGGTG | TAATCGAACTCACTGAGGGCACCGT |
| MyoD (mouse) | CTACCCAAGGTGGAGATCCTG | CACTGTAGTAGGCGGTGTCGT |

**Supplementary Table 2. Sequences of RNA and DNA oligonucleotides (Continued)**

| **Name** | **Sense strand/Sense primer (5'-3')** | **Antisense strand/Antisense primer (5'-3')** |
| --- | --- | --- |
| MyoG (mouse) | ATCCAGTACATTGAGCGCCTAC | GACGTAAGGGAGTGCAGATTGT |
| **gRNA for creating MPM-KO mice** | | |
| 1500011K16Rik-gRNA | TGGCGGACGTGTCTGAGAGG |  |
| **Primers for RACE** | | |
| **3’RACE** | | |
| 3’RACE-adaptor | ATGGCAGCAAGGTGATCACTAAAGTGATATCCTTTTTTTTTTTTTTTTVN | |
| 3’RACE-adaptor-primer | ATGGCAGCAAGGTGATCACTAAA |  |
| Nest PCR-primer (mouse) | GCAATGGCGGACGTGTCTGAGA |  |
| Nest PCR-primer (human) | GTTGTCCGTGCTAGTAGCCTTCGCTTC |  |
| **5’RACE** |  |  |
| 5’RACE-CDS-primer (mouse) | CTTGCAAGCCGTGCAAGGACGACTCC |  |
| Nest PCR-primer (mouse) | GATTACGCCAAGCTTGCGGCTCCCACGATCCG |  |
| 5’RACE-CDS-primer (human) | GAGTCTCAGGCCAGGTCCAGCTTCTTC |  |
| Nest PCR-primer (human) | GCAGCCTCCTTTTCCTCCAGTCCAAGT |  |
